# Is hand-wrist radiography still necessary in orthodontic treatment planning?

**DOI:** 10.1186/s12903-024-04396-2

**Published:** 2024-05-27

**Authors:** Musa Bulut, Yasin Hezenci

**Affiliations:** 1 Faculty of Dentistry, Department of Orthodontics, Bolu Abant Izzet Baysal University, Bolu, Turkey; 2 Diş Hekimliği Fakültesi, Ortodonti A.D. Gölköy Kampüsü, Bolu Abant İzzet Baysal Üniversitesi, Bolu, Turkey

**Keywords:** Cervical vertebrae maturation, Hand-wrist maturation, Growth spurt

## Abstract

**Objectives:**

The aim of our study is to compare the relationship between hand-wrist and cervical vertebra maturation stages with chronological age and to investigate the effect of malocclusion type on the relationship between these methods.

**Materials and methods:**

Hand-wrist and cephalometric radiographs of 1000 patients (526 females, 474 males) with a mean age of 13.41 ± 1.83 were analyzed. The methods of Bacetti et al. were used for the cervical vertebra maturation stage, and Björk, Grave and Brown’s methods were used for the hand-wrist maturation stage. One-way ANOVA test was applied to compare skeletal classes between them. Tukey post hoc test was used to determine the differences. The relationship between the malocclusion type, cervical vertebra and hand-wrist maturation stages was evaluated with the Spearman correlation test.

**Results:**

Spearman’s correlation coefficient was 0.831, 0.831 and 0.760 in Class I, II and III females, respectively. In males, it was calculated as 0.844, 0.889 and 0.906, respectively. When sex and malocclusion were not differentiated, the correlation was found to be 0.887. All were statistically significant (*P* < 0.001). The highest correlation was observed in class III males, while the lowest was found in class III females.

**Conclusion:**

Cervical vertebrae can be used safely to assess pubertal spurt without hand-wrist radiography. Diagnosing growth and development stages from cephalometric images is important in reducing additional workload and preventing radiation risk.

## Introduction

Dentofacial problems affect the jaws in different planes and their relations with each other. Class II malocclusions, one of the sagittal direction anomalies, are frequently encountered conditions, and it has been reported that it is observed between 10% and 40% in studies conducted in different societies [1–4]. Although it is mostly characterized by lower jaw retrognathia, cases where the upper jaw is prominent or a combination of the two conditions are also seen. Timing is the most crucial factor in treatment approaches that target growth modification of the mandible in the sagittal plane and stimulate mandible growth [5]. Determining the skeletal growth stage in the treatment planning of patients with Class II malocclusion is also very important in terms of the timing of functional orthopedic treatment, the type of appliance to be used and the duration of the treatment. It has also been reported that a significant increase in mandibular length can only be achieved when functional orthopedic treatment is performed in pubertal growth spurt or just after the growth spurt [6, 7].

However, while chronological age gives general information about the development of the individual, biological age stands out in the orthodontic treatment plan. Significant discrepancies between chronological and biological age have also been reported [8, 9]. Optimal timing for dentofacial orthopedics in orthodontic treatments is linked to the correct diagnosis of periods of accelerated growth critical to skeletal impact [10].

As in the medical field, radiological imaging systems have a significant place in the dental field for diagnosis and diagnosis. Cervical vertebral maturation (CVM) [11] and hand-wrist bones (HWM) [12, 13] are the most commonly used methods for determining the skeletal developmental stage. With the method developed by Björk, hand and wrist maturation can be determined in detail in 9 stages in a simple and easy way [14]. Some researchers have tried correlating developmental stages with skeletal features other than the hand and wrist to avoid an additional X-ray [15, 16].

It will be beneficial to determine the developmental stages from cephalometric radiographs, which are routinely taken from patients for cephalometric analysis and have an important place in orthodontic diagnosis. The patient will be protected from the additional radiation dose, and the physician’s workload will be reduced. CVM methods are effective in a growth spurt, evaluation of individual height growth, and mandibular growth estimation [17, 18]. In addition, studies have reported high correlations between the hand-wrist and CVM stages, and there is no difference between these methods in determining skeletal age [10, 11, 19–21].

Our hypothesis is that there is no difference between wrist maturation and cervical vertebra maturation, and that this situation is the same in different malocclusions. For this reason, there is no need for wrist radiographs. Therefore, our aim is to compare the relationship between hand-wrist and cervical vertebra maturation stages with chronological age and to investigate the effect of malocclusion type on the relationship between these methods.

## Materials and methods

In this study, hand-wrist and cephalometric radiographs of 1000 patients (526 females, 474 males) were randomly taken from the archives of Bolu Abant Izzet Baysal University Faculty of Dentistry Department of Orthodontics. Bolu Abant Izzet Baysal University Clinical Research and Ethics Committee approved the study and the informed consent was waived due to the retrospective nature of this study. Recordings taken on the same day and with the same machine (Pax Uni 3D; Vatech, Seoul, Korea) were selected. Only left-hand radiography was preferred for the wrist. The ages of the subjects ranged from 7 years 8 months to 17 years 10 months, with a mean age of 13.41 ± 1.83 (13.54 ± 1.84 years for males, 13.30 ± 1.83 years for females). The inclusion criteria for the study are listed below:

- Absence of congenital or acquired malformations in the cervical spine or wrist region.
- No systemic disease,
- Good radiographic image quality,
- Hand-wrist and cephalometric images were taken on the same day,
- Normal growth and development.

The methods described by Bacetti et al. [19] and McNamara and Franchi [20] were used for the CVM stage (Table 1), and the methods described by Björk, Grave, and Brown [22, 23] for the HWM stage (Table 2). Malocclusion classification was made according to the ANB value obtained by cephalometric analysis. If the ANB (˚) angle was between 0˚ and 4˚, it was recorded as skeletal Class I, if it was greater than 4˚, it was recorded as Class II, and if it was less than 0˚, it was recorded as Class III [24]. All evaluations were performed by the same researcher (Y. H.) in a dark room.

**Table 1:** The stages of cervical vertebrae maturation described by Bacetti et al. [18]

**Cervical Stage 1 (CS1).** The lower borders of all the three vertebrae (C2-C4) are flat. The bodies of both C3 and C4 are trapezoid in shape.
**Cervical Stage 2 (CS2).** A concavity is present at the lower border of C2. The bodies of both C3 and C4 are still trapezoid in shape.
**Cervical Stage 3 (CS3).** Concavities at the lower borders of both C2 and C3 are present. The bodies of C3 and C4 may be either trapezoid or rectangular horizontal in shape.
**Cervical Stage 4 (CS4).** Concavities at the lower borders of C2, C3, and C4 now are present. The bodies of both C3 and C4 are rectangular horizontal in shape.
**Cervical stage 5 (CS5).** The concavities at the lower borders of C2, C3, and C4 still are present. At least one of the bodies of C3 and C4 is squared in shape. If not squared, the body of the other cervical vertebra still is rectangular horizontal.
**Cervical Stage 6 (CS6).** The concavities at the lower borders of C2, C3, and C4 still are evident. At least one of the bodies of C3 and C4 is rectangular vertical in shape. If not rectangular vertical, the body of the other cervical vertebra is squared.

**Table 2:** The stages of skeletal maturation of hand-wrist radiographs according to the method of Björk, Grave, and Brown [20, 21]

**Stage 1 (PP2)**: Epiphysis of proximal phalanx of index finger (PP2) is same width as diaphysis
**Stage 2 (MP3)**: Epiphysis of middle phalanx of middle finger (MP3) is same width as diaphysis
**Stage 3 (Pisi-H1-R)**: Pisi, visible ossification of pisiform; H1: ossification of the hamular process of the hamatum; R, same width of epiphysis and diaphysis of radius
**Stage 4 (S-H2)**: S, first mineralization of ulnar sesamoid bone of metacarpophalangeal joint of hamatum; H2, progressive ossification of hamular process of hamatum
**Stage 5 (MP3cap-PP1cap-Rcap**): Diaphysis is covered by cap-shaped epiphysis; in MP3cap, process begins at middle phalanx of third finger; in PP1cap, at proximal phalanx of thumb; in Rcap, at Radius
**Stage 6 (DP3u)**: Visible union of epiphysis and diaphysis at distal phalanx of middle finger (DP3)
**Stage 7 (PP3u)**: Visible union of epiphysis and diaphysis at proximal phalanx of little finger (PP3)
**Stage 8 (MP3u)**: Union of epiphysis and diaphysis at middle phalanx of middle finger is clearly visible (MP3)
**Stage 9 (Ru)**: Complete union of epiphysis and diaphysis of radius

### Statistical analysis

Statistical package program SPSS V. 26.0 (Statistical Package for the Social Sciences, IBM, NY, USA) was used for data analysis. The sample size was calculated using the G*power 3.1.9.7 program (Heinrich-Heine-University, Düsseldorf, Germany) with a statistical power of 80%, the effect width determined according to Cohen as 0.20, and a significance level of 0.05, the required number of patients was determined as 788 [25]. Descriptive statistics were obtained for the individual mean ages of the HWM and CVM stages. According to the Kolmogorov-Smirnov test, CVM and HWM stages distribution with age was normal. One-way ANOVA test was applied to compare the skeletal classes between them. Tukey post hoc test was used to determine the differences. The relationship between HWM and CVM stages was evaluated by Spearman correlation test. The HWM and CVM classification of 30 randomly selected patients was repeated 1 month later by the same investigator to check for observer reliability. The measurement error was evaluated with the kappa test and the results were interpreted with the Landis and Koch method [26].

## Results

As a result of the analysis applied to evaluate the consistency between observations, Kappa values for HWM and CVM were significantly higher (Kappa coefficient: 0.914 and 1; *p* < 0.001;). Weighted kappa coefficients show an almost perfect agreement according to the Landis and Koch scales [26]. Descriptive data of maturation stages by sex (n, mean age, std.) are given in Table 3.

**Table 3:** Mean chronologic age and percentage distribution of CVM and HWM stages by sex

	Females				Males				Total	
Stages	n	%	Mean	Std.	n	%	Mean	Std.	n	%
CS1	12	2.30%	9.88	1.32	31	6.50%	11.3	1.62	43	4.30%
CS2	40	7.60%	10.91	1.14	111	23.40%	12.11	1.37	151	15.10%
CS3	62	11.80%	11.62	1.03	102	21.50%	13.03	1.16	164	16.40%
CS4	85	16.20%	12.58	1.17	120	25.30%	13.94	1.12	205	20.50%
CS5	226	43.00%	14.01	1.4	78	16.50%	15.61	1.03	304	30.40%
CS6	101	19.20%	14.73	1.28	32	6.80%	15.74	0.98	133	13.30%
S1	3	0.60%	9.56	1.91	24	5.10%	10.93	1.89	27	2.70%
S2	11	2.10%	9.85	1.12	45	9.50%	11.55	1.21	56	5.60%
S3	18	3.40%	10.64	1.12	66	13.90%	12	1.08	84	8.40%
S4	33	6.30%	11.29	0.92	70	14.80%	13.03	0.89	103	10.30%
S5	108	20.50%	11.97	1.12	134	28.30%	13.72	1.02	242	24.20%
S6	59	11.20%	12.77	0.77	36	7.60%	14.88	0.89	95	9.50%
S7	21	4.00%	13.39	0.89	10	2.10%	15.06	0.8	31	3.10%
S8	129	24.50%	13.88	1.08	49	10.30%	15.41	0.95	178	17.80%
S9	144	27.40%	15.13	1.16	40	8.40%	16.27	0.88	184	18.40%
Total	526	100			474	100			1000	100

The most common cervical vertebral maturation stages (CS) in females were CS5 (43%), CS6 (19.2%) and CS4 (16.2%), respectively. In males, CS4 (25.3%), CS2 (23.4%) and CS3 (21.5%) were observed, respectively (Table 3).

For the hand-wrist maturation stages (S), the most common ones were S9 (27.4%), S8 (24.5%) and S5 (20.5%) in females, S5 (28.3%), S4 (14.8%) and S3 in males, respectively. (13.9%) (Table 3).

Correlations between the hand-wrist and cervical vertebral maturation stages are shown in Table 4. Spearman’s correlation coefficient was 0.831, 0.831 and 0.760 in Class I, II and III females, respectively. In males, it was calculated as 0.844, 0.889 and 0.906, respectively. When sex and malocclusion were not differentiated, the correlation was found to be 0.887. All were statistically significant (*P* < 0.001). The highest correlation was observed in class III males, while the lowest was found in class III females.

**Table 4:** Distribution of maturation stages according to malocclusion type and correlation coefficients

		CVM																	
		**Sınıf I**						**Sınıf II**						**Sınıf III**					
HWM		CS1	CS2	CS3	CS4	CS5	CS6	CS1	CS2	CS3	CS4	CS5	CS6	CS1	CS2	CS3	CS4	CS5	CS6
S1	F	1	-	-	-	-	-	-	-	-	-	-	-	1	-	1	-	-	-
S2	F	3	3	-	-	-	-	1	3	1	-	-	-	-	-	-	-	-	-
S3	F	3	4	4	-	-	-	1	3	1	-	-	-	2	-	-	-	-	-
S4	F	-	8	9	4	-	-	-	6	4	-	-	-	-	1	-	1	-	-
S5	F	-	7	25	19	4	-	-	2	13	21	2	1	-	3	2	5	4	-
S6	F	-	-	-	16	23	1	-	-	1	9	6	1	-	-	-	1	-	1
S7	F	-	-	-	2	9	-	-	-	-	3	4	1	-	-	-	1	1	-
S8	F	-	-	1	-	58	14	-	-	-	-	38	11	-	-	-	2	4	1
S9	F	-	-	-	1	45	48	-	-	-	-	20	18	-	-	-	-	8	4
r		0.831*						0.831*						0.760*					
r		0.824*																	
S1	M	5	4	1	-	-	-	7	4	-	-	-	-	2	1	-	-	-	-
S2	M	7	20	3	-	-	-	3	5	-	-	-	-	2	4	-	1	-	-
S3	M	4	19	11	2	-	-	1	16	8	1	-	-	-	2	2	-	-	-
S4	M	-	13	16	12	-	-	-	12	11	5	-	-	-	-	-	1	-	-
S5	M	-	8	23	34	4	-	-	3	19	30	-	-	-	-	6	7	-	-
S6	M	-	-	2	12	3	2	-	-	-	5	12	-	-	-	-	-	-	-
S7	M	-	-	-	2	3	1	-	-	-	1	3	-	-	-	-	-	-	-
S8	M	-	-	-	5	16	6	-	-	-	1	15	2	-	-	-	-	3	1
S9	M	-	-	-	1	7	14	-	-	-	-	7	3	-	-	-	-	5	3
r		0.844*						0.889*						0.906*					
r								0.866*											

Combined r: 0.887*

*P < 0.001

† r, Correlation coefficient; F, female; M, male

There was no significant difference between males in any of the CVM and HWM stages of skeletal malocclusion type according to age. In females, significant differences were found in the CVM CS5 and HWM S3, S4, S6 and S9 stages according to malocclusion types (Table 5).

**Table 5:** Tukey post hoc test, in which significant differences were noted between malocclusion types in females

	Class	I		III	
		Mean D	*p*	Mean D	*p*
CVM CS5	II	-0.49^*^	0.04	-0.17	0.89
	III	− 0.33	0.63		
HWM S3	II	0.73	0.260	1.75	0.058
	III	2.49^*^	0.004		
HWM S4	II	-0.44	0.357	1.86^*^	0.020
	III	1.41	0.075		
HWM S6	II	-0.59^*^	0.019	0.69	0.422
	III	0.10	0.981		
HWM S9	II	-0.69^*^	0.005	0.67	0.170
	III	-0.02	0.998		

Tukey post hoc test

*, *P* < 0.05

## Discussion

Optimal timing for dentofacial orthopedics in orthodontic treatments is linked to the accurate diagnosis of accelerated growth periods critical to skeletal impact. Timing is the most crucial factor in treatment approaches targeted for growth modification [5, 10].

Therefore, our study was designed to evaluate the relationship between hand-wrist and cervical vertebra maturation stages with chronological age and determine the effect of malocclusion type, which has not been investigated before, on the relationship between these methods. In the studies, it was stated by the researchers that the growth spurt periods showed racial differences [27, 28]. Many studies report that growth and development periods differ according to sex [29, 30]. Similar to previous studies, our study found that females complete growth spurt before males. It was found that pubertal spurt started at a mean age of 13.03 in males and 11.29 in females. The mean age at the end of the spurt was 14.88 years in males and 12.77 years in females.

In a study, skeletal and dental maturation stages and growth tendencies of individuals with Class III malocclusion were analyzed [31]. In another study, CVM maturation stages were examined for the different malocclusion types [32]. However, no study has been found in which sagittal dimension anomalies are included for the HWM and CVM stages when skeletal development is evaluated and whether the skeletal class relationship affects the developmental stage.

Armond et al. [32], investigated the relationship between CVM stage and malocclusion types according to age and sex. They reported differences in maturation stages between the sexes in the same age group. Although they found the relationship between the CVM stage and malocclusion type significant, they did not observe any difference between individuals with Class I and III malocclusion. They found that skeletal maturation occurred later in males with Class II malocclusion. In our study, the stages were analyzed in detail, and the mean age was significantly higher in class II CS5 females. In addition, significant differences were observed in females in some of the HWM stages. No difference was observed between the types of malocclusion in male individuals at any stage. The differing findings of the studies may have resulted from the ethnicity of the individuals included and the number of individuals included. In addition, the significant differences observed in females can be explained by the effects of hormones being higher in females, and they complete their developmental stages earlier [33].

Like our study, many researchers found statistically significant correlations between HWM and CWM [27, 34–36]. The fact that the number of individuals included in our study is high and that many studies in the literature support it shows that it is optional to obtain diagnostic hand-wrist radiographs from patients.

In this study, we included a large sample group with a wide age range with skeletal Class I, Class II and Class III malocclusions in the prepubertal, pubertal and postpubertal stages in both sexes. This situation directly affects treatment planning and methods. Cephalometric films, which are already used in orthodontic diagnosis to determine skeletal growth periods in dentofacial orthopedics, will allow patients to be exposed to lower doses of radiation and reduce the workload in the clinical practice.

A limitation of our study is that there may be individual differences in skeletal maturation due to hereditary factors and environmental factors such as race, physical activity level, and nutrition. In addition, since this study examined patients who applied to the orthodontic clinic and already had malocclusion, more comprehensive research is required to make assumptions about the general population. Additional radiation dose is received for hand-wrist films. Also, artifacts can be found on radiographs of young children in the early stages of skeletal development [37]. CVM method has a low repeatability between different observers when classifying the shape differences of vertebral bodies [38]. Also, the duration between the CVM phases cannot be fully understood [39].

## Conclusion

A high correlation was found between the hand-wrist and cervical vertebra maturation stages. The highest correlation was observed in class III males, while the lowest was found in class III females.

Due to the differences observed in females according to malocclusion types at different stages of skeletal maturation, more careful consideration should be given to growth assessment.

The findings suggest that the CVM method can be used for pubertal growth spurt assessment without hand-wrist radiography. Diagnosis of growth and development stages from cephalometric images is important in reducing the additional workload and the amount of radiation received.

## Data Availability

The datasets used during the current study available from the corresponding author on reasonable request.
